# The COUGHVID crowdsourcing dataset, a corpus for the study of large-scale cough analysis algorithms

**DOI:** 10.1038/s41597-021-00937-4

**Published:** 2021-06-23

**Authors:** Lara Orlandic, Tomas Teijeiro, David Atienza

**Affiliations:** Embedded Systems Laboratory (ESL), EPFL, Lausanne, 1015 Switzerland

**Keywords:** Respiratory signs and symptoms, Diagnostic markers

## Abstract

Cough audio signal classification has been successfully used to diagnose a variety of respiratory conditions, and there has been significant interest in leveraging Machine Learning (ML) to provide widespread COVID-19 screening. The COUGHVID dataset provides over 25,000 crowdsourced cough recordings representing a wide range of participant ages, genders, geographic locations, and COVID-19 statuses. First, we contribute our open-sourced cough detection algorithm to the research community to assist in data robustness assessment. Second, four experienced physicians labeled more than 2,800 recordings to diagnose medical abnormalities present in the coughs, thereby contributing one of the largest expert-labeled cough datasets in existence that can be used for a plethora of cough audio classification tasks. Finally, we ensured that coughs labeled as symptomatic and COVID-19 originate from countries with high infection rates. As a result, the COUGHVID dataset contributes a wealth of cough recordings for training ML models to address the world’s most urgent health crises.

## Abstract (summary)

**Table:** 

Measurement(s)	Cough
Technology Type(s)	Microphone Device
Factor Type(s)	COVID-19 status • location • age • gender • respiratory condition
Sample Characteristic - Organism	Homo sapiens

Machine-accessible metadata file describing the reported data: 10.6084/m9.figshare.14377019

## Background & Summary

The novel coronavirus disease (COVID-19), declared a pandemic by the World Health Organization on March 11, 2020, has claimed over 2.5 million lives worldwide as of March 2021^1^. Epidemiology experts concur that mass testing is essential for isolating infected individuals, contact tracing, and slowing the progression of the virus^2,3^. While advances in testing have made these tools more widespread in recent months, there remains a need for inexpensive, rapid, and scalable COVID-19 screening technology^4^.

One of the most common symptoms of COVID-19 is a dry cough, which is present in approximately 67.7% of cases^5^. Cough sound classification is an emerging field of research that has successfully leveraged signal processing and artificial intelligence (AI) tools to rapidly and unobtrusively diagnose respiratory conditions like pertussis^6^, pneumonia and asthma^7^ using nothing more than a smartphone and its built-in microphone. Several research groups have begun developing algorithms for diagnosing COVID-19 from cough sounds^8,9^. One such initiative, AI4COVID^8^, provides a proof-of-concept algorithm but laments the lack of an extensive, labeled dataset that is needed to effectively train AI algorithms.

There are several existing COVID-19 cough sound datasets used to train Machine Learning (ML) models. Brown *et al*.^9^ have amassed a crowdsourced database of more than 10,000 cough samples from 7,000 unique users, 235 of which claim to have been diagnosed with COVID-19. However, the authors have not automated the data filtering procedure and consequently needed to endure the time-consuming process of manually verifying each recording. Furthermore, this dataset is not yet publicly available and therefore cannot be used by other teams wishing to train their ML models. The Coswara project^10^, on the other hand, has publicly provided manual annotations of the crowdsourced COVID-19 coughs they have received, but as of March 2021, their dataset contains only 1,500 samples. An alternative approach to crowdsourcing is the NoCoCoDa^11^, a database of cough sounds selected from media interviews of COVID-19 patients. However, this database only includes coughs from 10 unique subjects, which is not enough for AI algorithms to successfully generalize to the global population.

In this work, we present the COUGHVID crowdsourcing dataset, which is an extensive, publicly-available dataset of cough recordings. With more than 25,000 recordings – 1,155 claiming to have COVID-19 – originating from around the world, it is the largest known public COVID-19-related cough sound dataset in existence. In addition to publicly providing most of our cough corpus, we have trained and open-sourced a cough detection ML model to filter non-cough recordings from the database. This automated cough detection tool assists developers in creating robust applications that automatically remove non-cough sounds from their databases. Furthermore, our open-sourced cough detection model, preprocessing methods, cough segmentation algorithm, and SNR estimation code enable the research community to seamlessly integrate their own datasets with COUGHVID while keeping the data processing pipeline consistent.

We have undergone an additional layer of validation whereby four expert physicians annotated a fraction of the dataset to determine which crowdsourced samples realistically originate from COVID-19 patients. In addition to COVID-19 diagnoses, our expert labels and metadata provide a wealth of insights beyond those of existing public cough datasets. These datasets either do not provide labels or contain a small number of samples. For example, the Google Audio Set^12^ contains 871 cough sounds, but it does not specify the diagnoses or pathologies of the coughs. Conversely, the IIIT-CSSD^13^ labels coughs as wet vs dry and short-term vs long-term ailments, but it only includes 30 unique subjects. The COUGHVID dataset publicly contributes over 2,800 expert-labeled coughs, all of which provide a diagnosis, severity level, and whether or not audible health anomalies are present, such as dyspnea, wheezing, and nasal congestion. Using these expert labels along with participant metadata, our dataset can be used to train models that detect a variety of participants’ information based on their cough sounds. Overall, our dataset contains samples from a wide array of participant ages, genders, COVID-19 statuses, pre-existing respiratory conditions, and geographic locations, which potentially enable ML models to successfully perform generalization.

Finally, we ensure that samples labeled as COVID-19 originate from countries where the virus was prevalent at the time of recording, and we perform an evaluation of the quality of the recordings containing cough sounds. The first step to building robust AI algorithms for the detection of COVID-19 from cough sounds is having an extensive dataset, and the COUGHVID dataset effectively meets this pressing global need.

## Methods

### Data collection

All of the recordings were collected between April 1st, 2020 and December 1st, 2020 through a Web application deployed on a private server located at the facilities of the École Polytechnique Fédérale de Lausanne (EPFL), Switzerland. The application was designed with a simple workflow and following the principle “one recording, one click”, according to which if someone simply wants to send a cough recording, they should have to click on no more than one item.

The main Web interface has just one “Record” button that starts recording audio from the microphone for up to 10 seconds. Once the audio recording is completed, a small questionnaire is shown to get some metadata about the age, gender, and current condition of the user, but even if the questionnaire is not filled, the audio is sent to the server. The variables captured in the questionnaire are described in Table 1. Also, the user is asked for permission to provide their geolocation information, which is not mandatory. Finally, since coughing is a potentially dangerous activity in the scope of a global pandemic, we provide easy-to-follow safe coughing instructions, such as coughing into the elbow and holding the phone at arm’s length, that can be accessed from the main screen.

**Table 1:** Metadata variables, as they appear in the JSON files.

Name	Mandatory	Range of possible values	Description
datetime	Yes	UTC date and time in ISO 8601 format	Timestamp of the received recording.
cough_detected	Yes	Floating point in the interval [0, 1]	Probability that the recording contains cough sounds, according to the automatic detection algorithm described in the Methods section.
SNR	Yes	Floating point in the interval [0, ∞)	An estimation of the signal-to-noise ratio of the cough recording.
latitude	No	Floating point value	Self-reported latitude geolocation coordinate with reduced precision.
longitude	No	Floating point value	Self-reported longitude geolocation coordinate with reduced precision.
age	No	Integer value	Self-reported age value.
gender	No	{female, male, other}	Self-reported gender.
respiratory_condition	No	{True, False}	The patient has other respiratory conditions (self-reported).
fever_muscle_pain	No	{True, False}	The patient has fever or muscle pain (self-reported).
status	No	{COVID, symptomatic, healthy}	The patient self-reports that has been diagnosed with COVID-19 (COVID), that has symptoms but no diagnosis (symptomatic), or that is healthy (healthy).
expert_labels_{1,2,3}	No	JSON dictionary with the manual labels from expert 1, 2 or 3	The expert annotation variables are described in Table 4.

### Database cleaning

A common pitfall of crowdsourced data is that it frequently contains samples unrelated to the desired content of the database. In order to allow users of the COUGHVID dataset to quickly exclude non-cough sounds from their analyses, we developed a classifier to determine the degree of certainty to which a given recording contains a cough sound. These output probabilities of the classifier were subsequently included in the metadata of each record under the cough_detected entry. The recordings determined by the ML model to be non-cough sounds are included in the database and can be utilized by developers to enhance the robustness of their applications, such as alerting users when they do not upload a valid cough sound. Developers may use these samples to train their own classifiers to detect specific sounds, such as breathing or wheezing, and create many other possible applications.

To train the classifier, we employed similar procedures to those of state-of-the-art cough classification models^14^. We first randomly selected a set of 215 recordings from our database. Then, we manually classified each recording as a cough sound if at least one cough was present, otherwise listing it as a non-cough sound. In the case that a recording contained both cough and non-cough audio events, the recording was discarded and a new one was randomly selected from the database. This process resulted in a nearly balanced sample of 121 cough sounds and 94 non-cough sounds including speaking, laughing, silence, and miscellaneous background noises. These recordings were preprocessed by lowpass filtering (*f*_*cutoff*_ = 6 kHz) and downsampling to 12 kHz. Next, 68 audio features commonly used for cough classification were extracted from each recording. The details of these state-of-the-art features are listed in Table 2. The power spectral density (PSD) feature was computed using 8 hand-selected frequency bands similar to those selected by Alvarez *et al*.^14^. These bands were chosen by analyzing the PSDs of cough vs non-cough signals and selecting the frequency ranges with the highest variation between the two classes.

**Table 2:** Cough Classifier Features.

Feature Class	Domain	Count	Computation Parameters
MFCC^6^	Mel Frequency	26	Mean and St. Dev of 13 MFCCs over time
EEPD^32^	Time	19	BPF intervals in 50-1000 Hz; See Chatrazzin *et al*.^32^ for details
Power Spectral Density^14^	Frequency	8	Frequency bands (Hz): 0-200, 300-425, 500-650, 950-1150, 1400-1800, 2300–2400, 2850–2950, 3800–3900
RMS Power^6^	Time	1	None
Zero Crossing Rate^6^	Time	1	None
Crest Factor^6^	Time	1	None
Recording Length	Time	1	None
Dominant Frequency^6^	Frequency	1	None
Spectral Centroid^6,33^	Frequency	1	None
Spectral Rolloff ^6,33^	Frequency	1	None
Spectral Spread^6,33^	Frequency	1	None
Spectral Skewness^6,33^	Frequency	1	None
Spectral Kurtosis^6,33^	Frequency	1	None
Spectral Bandwidth^33^	Frequency	1	None
Spectral Flatness^6,33^	Frequency	1	None
Spectral St. Dev^6,33^	Frequency	1	None
Spectral Slope^6,33^	Frequency	1	None
Spectral Decrease^6,33^	Frequency	1	None

We developed an eXtreme Gradient Boosting (XGB)^15^ classifier to perform the cough classification based on audio features. In order to assess the performance of the model across various sets of testing and training participants, we employed a nested k-fold cross-validation (CV) procedure^16^. First, the dataset was partitioned into 10 segments, each with an equal number of recordings, hereinafter known as the “outer CV folds”. In each outer fold, one segment was used for testing and the rest were used for training. Then, within each outer CV fold, the XGB hyperparameters were tuned using Tree-structured Parzen Estimators (TPE)^17^. TPE works by further partitioning the training data into 10 segments, known as the “inner CV folds”, and then determining which set of hyperparameters exhibits the highest mean precision across the 10 folds. Following each TPE procedure, we tested the tuned XGB model using the testing data of its respective outer CV fold and computed several accuracy metrics. We then averaged each accuracy metric across the 10 outer CV folds and recorded its mean and standard deviation, as displayed in Table 3. We can see that this cough classifier exhibits a mean AUC of 96.4% and a standard deviation of only 3.3%, indicating that the model performs well and shows little variance among different testing groups. To develop a final, usable model to share with the research community, we reassembled the entire dataset and randomly selected 22% of the recordings for assessing the generalization capabilities of our model to unseen test participants. The remainig 78% of the data was used to train the final XGB classifier and determine one final set of hyperparameters using the aforementioned TPE cross-validation procedure, this time using a 5-fold random permutation-based ShuffleSplit CV^18^.

**Table 3:** Cough Classifier Performance (cough_detected threshold = 0.8).

Metric	CV Mean ± St. Dev	Final Model
Precision	95.4 ± 7.1	95.5
Sensitivity	78.2 ± 10	80.8
Specificity	95.3 ± 8.4	95.5
Balanced Accuracy	86.7 ± 3.9	88.1
AUC	96.4 ± 3.3	96.5

**Fig. 1:**
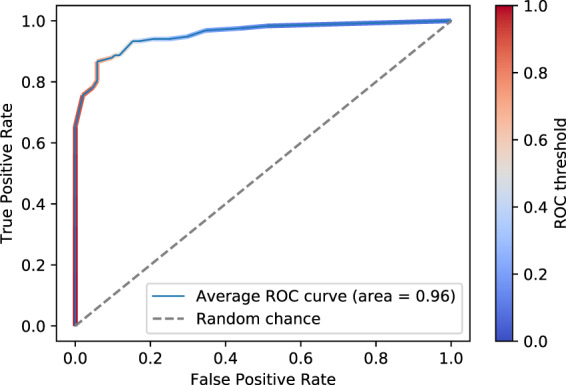
Averaged receiver operating characteristic curve across the 10 cross-validation folds of the cough classifier.

The ROC curve of the cough classifier, which shows the mean true positive rate versus the mean false positive rate of the testing results across the 10 outer CV folds, is displayed in Fig. 1. Users of the COUGHVID database can consult this figure to set a cough detection threshold that suits their specifications. We recommend using a cough_detected value of 0.8 because, as shown in Fig. 1 and in Table 3, this threshold exhibits an average precision of 95.4%. Therefore, only 4.6% of recordings with a cough_detected probability greater than 0.8 can be expected to contain non-cough events, which is not a large enough portion of the dataset to significantly bias cough classification algorithms. Similarly, the final model exhibits a precision of 95.5% and sensitivity of 80.8%, indicating that the model successfully removes most non-cough recordings while maintaining over 80% of recordings that truly contain coughs. Finally, the samples confirmed by experts to contain coughs have a cough_detected value of 1.0, whereas those that experts noted did not contain coughs (less than 3% of the annotated data) have a cough_detected value of 0.

### Expert annotation

To enhance the quality of the dataset with clinically validated information, we were assisted by four expert physicians. Each of them revised 1000 recordings, selecting one of the predefined options to each of the following 10 items:

1. **Quality**: Good; Ok; Poor; No cough present.
2. **Type of cough**: Wet (productive); Dry; Can’t tell.
3. **Audible dyspnea**: Checkbox.
4. **Audible wheezing**: Checkbox.
5. **Audible stridor**: Checkbox.
6. **Audible choking**: Checkbox.
7. **Audible nasal congestion**: Checkbox.
8. **Nothing specific**: Checkbox.
9. **Impression: I think this patient has…**: An upper respiratory tract infection; A lower respiratory tract infection; Obstructive lung disease (Asthma, COPD, …); COVID-19; Nothing (healthy cough).
10. **Impression: the cough is probably…**: Pseudocough/Healthy cough (from a healthy person); Mild (from a sick person); Severe (from a sick person); Can’t tell.

As a labeling support tool, we used an online spreadsheet using Google Sheets^©^. Thus, the experts could play the recordings directly inside the browser and select their answers in a convenient way. Once a recording had been labeled, the background color of the full row turned to green for easier navigation. The time required for each expert for labeling the 1000 recordings was around 10 hours, without significant differences among the four experts.

In addition to the personal spreadsheets, the experts were provided with the following general instructions:

- *For binary variables (Columns E-J) the box should be ticked for any reasonable suspicion of the sounds being heard*.
- *In Column K (“Impression: I think this patient has…”), you should check what you consider the most likely diagnosis, knowing that the records were collected between 01/04/2020 and 01/12/2020*.
- *Each row is automatically marked as “completed” after providing an answer to column K (question number 9). However, please try to give an answer to every column*.

Also, the following criteria for assessing quality was indicated to the experts:

- **Good**: *Cough present with minimal background noise*.
- **Ok**: *Cough present with background noise*.
- **Poor**: *Cough present with significant background noise*.
  The selection of the recordings to be labeled was done through stratified random sampling and after pre-filtering using the automatic cough classifier described above, requiring a minimum probability of 0.8 of containing cough sounds. The distribution of recordings labeled by each expert was based on the self-reported status variable, as follows:
  - 25% of the recordings with COVID value.
  - 35% of the recordings with symptomatic value.
  - 25% of the recordings with healthy value.
  - 15% of the recordings with no reported status.

This stratification ensured that between the four experts, 100% of the recordings labeled as COVID-19 by users that have a cough_detected value above 0.8 were labeled by at least one expert. Finally, we ensured that 15% of the recordings were labeled by all three reviewers, so that we could assess the level of agreement among them.

### Ethical compliance

All of the data collection and annotation was done in compliance with relevant ethical regulations. Informed consent was obtained by all participants who uploaded their cough sounds and metadata to our servers.

## Data Records

All of the publicly-available data records are stored on a Zenodo repository^19^. Considering the recordings with a cough_detected value greater than 0.8, as explained in the Database Cleaning section, the database consists of nearly 35 hours of audio samples. This corresponds to approximately 37,000 segmented coughs, as determined using the segmentation procedure described in the Recording Quality Estimation subsection. Each cough recording consists of two files with the same name but different extensions. One of the files contains the audio data, as it was directly received at the COUGHVID servers, and it can be in the WEBM^20^ or OGG^21^ formats, respectively with the .webm and .ogg extensions. In all cases, the audio codec is Opus^22^, with a sampling frequency of 48 kHz and operating in variable bitrate (VBR) mode. For more than 40% of the recordings, the effective bitrate is 48 kbit/s, which is in the highest bandwidth range for mono recordings^22^. For the rest of the recordings, the reason for having a lower effective bitrate is the presence of long periods of silence, which is in fact desirable given the application. The second file contains the metadata encoded as plain text in the JSON format^23^, and has the .json extension. The file name is a random string generated according to the UUID V4 protocol^24^.

In the metadata we may distinguish three types of variables, related to: (1) context information (timestamp and the probability that the recording actually contains cough sounds), (2) self-reported information provided by the user, and (3) the labels provided by expert medical annotators about the clinical assessment of the cough recording. The only processing performed on the metadata was the reduction in the precision of the geolocation coordinates to just one decimal digit to ensure privacy protection. A full description of all the metadata variables is provided in Tables 1 and 4.

**Table 4:** Variables provided by the expert annotators.

Name	Range of possible values	Description
quality	{good, ok, poor, no_cough}	Quality of the recorded cough sound.
cough_type	{wet, dry, unknown}	Type of cough.
dyspnea	{True, False}	Audible dyspnea.
wheezing	{True, False}	Audible wheezing.
stridor	{True, False}	Audible stridor.
choking	{True, False}	Audible choking.
congestion	{True, False}	Audible nasal congestion.
nothing	{True, False}	Nothing specific is audible.
diagnosis	{upper_infection, lower_infection, obstructive_disease, COVID-19, healthy_cough}	Impression of the expert about the condition of the patient. It can be an upper or lower respiratory tract infection, an obstructive disease (Asthma, COPD, etc), COVID-19, or a healthy cough.
severity	{pseudocough, mild, severe, unknown}	Impression of the expert about the severity of the cough. It can be a pseudocough from a healthy patient, a mild or severe cough from a sick patient, or unknown if the expert can’t tell.

As an illustrative example, let us consider the recording with UUID 4e47612c-6c09-4580-a9b6-2eb6bf2ab40c depicted in Box 1. We can see the audio properties using a tool such as ffprobe (included in the ffmpeg software package^25^), while the metadata can be directly displayed as a text file.

For convenience, a file compiling all of the available metadata is also provided. This file is named metadata_compiled.csv, and is a CSV file with 51 columns and one row per record. The first column corresponds to the UUID of each recording, and may be used as an index, while the rest of the columns correspond to the variables described in Tables 1 and 4, plus the SNR column described below in the “Recording Quality Estimation” section. The expert annotation variables have been expanded, and are named quality_1, cough_type_1 … diagnosis_4, severity_4.

Box 1 **4e47612c-6c09-4580-a9b6-2eb6bf2ab40c.webm**

Input #0, matroska,webm, from ‘4e47612c-6c09-4580-a9b6-2eb6bf2ab40c.webm’:

      Metadata:

            encoder:                              Chrome

      Duration: N/A, start: 0.000000, bitrate: N/A

            Stream #0:0(eng): Audio: opus, 48000 Hz, mono, fltp (default)

**4e47612c-6c09-4580-a9b6-2eb6bf2ab40c.json**

{

            “datetime”: “2020-04-10T10:30:31.576207 + 00:00”,

            “cough_detected”: “0.9466”,

            “age”: “50”,

            “gender”: “male”,

            “respiratory_condition”: “True”,

            “fever_muscle_pain”: “False”,

            “status”: “COVID-19”,

            “expert_labels_1”: {

                        “quality”: “ok”,

                        “cough_type”: “dry”,

                        “dyspnea”: “False”,

                        “wheezing”: “False”,

                        “stridor”: “False”,

                        “choking”: “False”,

                        “congestion”: “False”,

                        “nothing”: “True”,

                        “diagnosis”: “COVID-19”,

                        “severity”: “mild”

            }

{

## Technical Validation

### Demographic representativeness

An important requirement for large datasets is that they must represent a wide range of participant demographics. Demographic statistics were collected across all recordings in our public dataset that provided metadata and that were classified as coughs with a probability above 0.8 by the XGB cough detection model. There were slightly more male participants than female (66.0% and 33.5%, respectively), and the majority of participants did not have pre-existing respiratory conditions (83.6%). The percentages of healthy, COVID-19 symptomatic, and COVID-19 positive participants were 78%, 16.1%, and 5.8%, respectively. The average age of the recordings was 36.5 years with a standard deviation of 13.9 years. This shows that a wide variety of ages, genders, and health statuses are captured within our dataset.

### Geographic representativeness

In order to assess the plausibility that samples labeled as COVID-19 truly originated from people who tested positive for the disease, we analyzed the geographic locations of the samples in our public dataset for which this information was provided. We then evaluated the COVID-19 statistics of the countries at the time each recording was sent to determine if these countries had high infection rates at the time of recording. Of the 14,787 samples that the XGB model classified as cough sounds, 8887 of them provided GPS information, 1014 of which reported COVID-19 symptoms and 410 claim to have been diagnosed with COVID-19. An analysis of this geodata revealed that our dataset contains recordings originating from 125 unique countries, reflecting the diversity of the dataset.

Studies have shown that coughing persists in a significant proportion of COVID-19 cases 14-21 days after receiving a positive PCR test^26^. Therefore, we combined the World Health Organization’s statistics on daily new COVID-19 cases^1^ with the United Nations 2019 population database^27^ to determine the rate of new infections in the country from which each recording originated in the 14 days prior to it being uploaded to our web server. This analysis revealed that 94.9% of our recordings labeled as COVID-19 came from countries with more than 20 newly-confirmed COVID-19 cases per 1 million people. Similarly, 93.1% of recordings labeled as symptomatic originated from these countries.

**Fig. 2:**
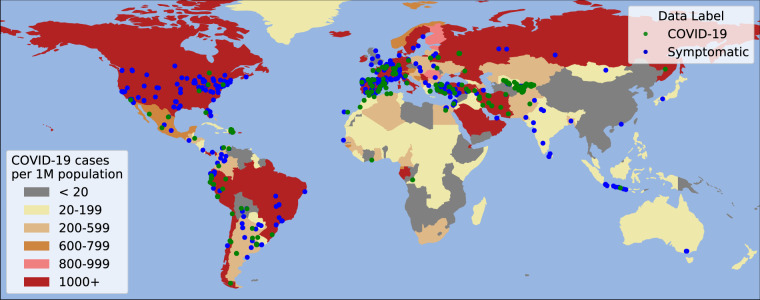
Cumulative COVID-19 cases in April and May 2020 per 1 million population, along with the GPS coordinates of the received recordings.

Figure 2 shows a map of the world with countries color coded according to the cumulative COVID-19 positive tests in April and May 2020 per 1 million population. We also show the COVID-19 and symptomatic recordings in our public corpus providing GPS information that were collected within this time period. This figure shows that most recordings were sent from countries with moderate-to-high COVID-19 infection rates at the time.

### Inter-rater reliability

In order to determine the extent to which the four expert physicians agreed on their cough sound labeling, Fleiss’ Kappa scores^28^, *K*_*Fleiss*_, were computed for each question among the 130 common recordings in the public dataset. *K*_*Fleiss*_ is a standard method for gauging the consistency of categorical labels across multiple reviewers. This measure ranges from −1 – indicating no agreement among any reviewers – to 1, corresponding to perfect agreement among all reviewers. The results are displayed in Table 5, which depicts the *K*_*Fleiss*_ measures along with the strength of agreement according to the benchmark scale proposed by Landis and Koch^29^. This analysis revealed moderate agreement on audible nasal congestion, fair agreement on the type of cough, as well as slight agreement on the cough severity, nothing specific, audible wheezing, and diagnosis.

**Table 5:** Inter-Expert Label Consistency.

Item	*K*_*Fleiss*_	Agreement^29^
quality	−0.06	Poor
cough_type	0.26	Fair
dyspnea	−0.02	Poor
wheezing	0.06	Slight
stridor	−0.01	Poor
choking	−0.01	Poor
congestion	0.41	Moderate
nothing	0.13	Slight
diagnosis	0.07	Slight
severity	0.15	Slight

There was a slight agreement among experts on the cough diagnosis (*K*_*Fleiss*_ = 0.07), which is reflective of the fact that COVID-19 symptomology includes symptoms of both upper respiratory tract infections (e.g., rhinorrhea, sore throat, etc.) and lower respiratory tract infections (e.g., pneumonia, ground-glass opacities, etc.)^30^. When the expert labels that are not “COVID-19” or “healthy cough” are changed to “other”, the agreement slightly increases (*K*_*Fleiss*_ = 0.08). To determine the commonalities in labels between individual experts, we computed *K*_*Fleiss*_ on the cough diagnoses of pairs of experts. There was a fair agreement between Experts 3 and 4 (*K*_*Fleiss*_ = 0.22), a slight agreement between Experts 1 and 4 (*K*_*Fleiss*_ = 0.04), and a slight agreement between Experts 1 and 2 (*K*_*Fleiss*_ = 0.07). Other pairs of experts exhibited poor agreement. While a lack of a clear diagnostic consensus between all experts is to be expected for a novel pathology, it is advisable that users take this into consideration when using the experts’ labels as ground-truth.

### Trends in expert COVID-19 cough labeling

All of the coughs in the public database that were labeled as COVID-19 among the four experts were subsequently pooled together and analyzed for trends in the attributes of the cough recordings. The vast majority of coughs do not exhibit audible dyspnea (93.72%), wheezing (92.43%), stridor (98.71%), choking (99.20%), or nasal congestion (99.03%). Additionally, 86.96% of COVID-19-labeled coughs are annotated as dry, which is consistent with literature stating that a dry cough is a common COVID-19 symptom^5,30^. Finally, 83.58% of these coughs are labeled as mild. These commonalities among COVID-19 labeled coughs reflect the consistency of the database.

### Recording quality estimation

Every user who uploaded their cough sound to the COUGHVID dataset presumably used a different device, potentially introducing a variation in recording quality due to the different recording hardware and software of each device. Furthermore, the recordings were captured at various locations around the world with non-constant degrees of background noise. In order to assist users of the COUGHVID dataset in estimating the quality of each signal, we provide open-sourced code to estimate the Signal-to-Noise Ratio (SNR) of each cough recording.

**Fig. 3:**
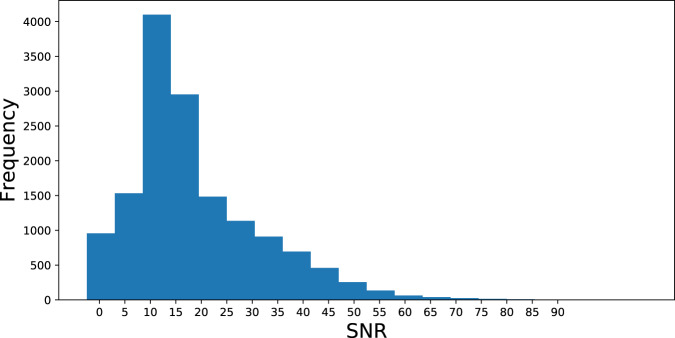
Histogram of estimated SNRs of every recording in the database with a cough_detected value greater than 0.8.

The SNR estimation method begins with a simple cough segmentation algorithm based on a digital hysteresis comparator on the signal power. The signal is first normalized to the [−1, 1] range, lowpass filtered (*f*_*cutoff*_ = 6 kHz), and downsampled to 12 kHz. Then, the hysteresis comparator identifies regions of the signal with rapid spikes in power, which is customary for cough sounds. Since the expiratory phase of the cough lasts 230-550 ms^31^, we discard any cough sounds lasting less than 200 ms. Furthermore, we consider the 200 ms before and after a cough sound as part of the cough, as this period may comprise the low-amplitude compressive and expiratory phases of the cough sound^31^. Following the cough segmentation, the SNR is computed as following Eq. 1,

1 $$SNR=20\times {log}_{10}\left(\frac{\sqrt{\frac{1}{|{x}_{cough}|}\sum _{x(n)\varepsilon {x}_{cough}}x{(n)}^{2}}}{\sqrt{\frac{1}{|{x}_{noise}|}\sum _{x(n)\varepsilon {x}_{noise}}x{(n)}^{2}}}\right)$$

where *x*_*cough*_ are the signal samples determined through segmentation to be part of a cough, and *x*_*noise*_ are all other signal samples, presumed to be background noise. Intuitively, this formula compares the signal power of the cough portion of the signal to that of the background noise. This SNR information is included under the SNR column of the recording metadata CSV file. A histogram of all SNR values for recordings with a cough_detected probability greater than 0.8 is shown in Fig. 3, showing a wider variety of signal qualities present in the dataset. Users of the database may employ these computed SNR values to filter the dataset by maintaining only recordings of a desired quality.

## Private Set and Testing Protocol

In order to ensure the reproducibility of the experimental results that use the COUGHVID crowdsourcing dataset, a private test set of 625 cough recordings has been kept out from publishing. Recordings in this private set have been randomly selected from those having at least labels from one expert. Tens of non-cough samples have been included in the test set (labeled with a cough_detected probability of 0) to assist developers in assessing the robustness of their models.

The evaluation of models on the private test set will be open to the entire scientific community, but to ensure a fair use, the performance measurements will be obtained by an independent evaluator. Any researcher that demonstrates promising results on the public dataset using cross-validation may apply for an independent evaluation on the private test set according to the protocol described in the data repository^19^. This protocol shall be regularly updated in line with the available technology to ensure that it is as convenient as possible for all parties.

Since one of the aims of this project is to go beyond the study of COVID-19, every variable except datetime and cough_detected may be considered the target of a prediction model. This opens the possibility to study several different problems within the same dataset, from just cough identification and sound quality assessment to the detection of different conditions, or even the estimation of age or gender. Conversely, a prediction model may require as input not just the sound recording, but also other metadata variables to provide the necessary context, such as the age or gender of the participant. The restrictions on the sets of variables that can be used as input or as a result of the algorithms will be kept to a minimum.

## Data Availability

The aforementioned XGB classifier used to remove non-cough recordings, feature extraction source code, cough preprocessing methods, cough segmentation function, and SNR estimation algorithm are available on our public repository.
